# Using acute tryptophan depletion to investigate predictors of treatment response in adolescents with major depressive disorder: study protocol for a randomised controlled trial

**DOI:** 10.1186/s13063-018-2791-4

**Published:** 2018-08-10

**Authors:** Richard M. Stewart, Sean D. Hood, Pradeep Rao, Julia K. Moore, Kevin C. Runions, Susannah E. Murphy, Janice W. Y. Wong, Florian D. Zepf

**Affiliations:** 1 Centre & Discipline of Child and Adolescent Psychiatry, Psychosomatics and Psychotherapy, Divisions of Paediatrics and Psychiatry, UWA Medical School, Faculty of Health and Medical Sciences, The University of Western Australia, 35 Stirling Highway (M561), Crawley WA, Perth, 6009 Australia; 2 Division of Psychiatry, UWA Medical School, Faculty of Health and Medical Sciences, The University of Western Australia, Perth, Australia; 3 Community Child and Adolescent Mental Health Services (CAMHS), Department of Health in Western Australia, Perth, Australia; 4 Paediatric Consult-Liaison, Acute Child and Adolescent Mental Health Services (CAMHS), Department of Health, Perth, Western Australia Australia; 5 Telethon Kids Institute, Perth, Australia; 6 Department of Psychiatry, Warneford Hospital, University of Oxford, Oxford, UK; 7 Specialised Child and Adolescent Mental Health Services (CAMHS), Department of Health in Western Australia, Perth, Australia

**Keywords:** Depression, Children, Adolescents, Serotonin, Tryptophan depletion, Pharmacology, Mood disorders, Neuroscience

## Abstract

**Background:**

Selective serotonin reuptake inhibitors (SSRIs) are amongst the most prescribed antidepressants for adolescents with depressive symptoms and major depressive disorder. However, SSRIs have significant shortcomings as a first-line treatment considering that not all patients respond to these antidepressants. Amongst paediatric populations, meta-analyses indicate that up to approximately 40% of patients do not respond, and for those who do show benefit, there is substantial heterogeneity in response onset. The neurotransmitter serotonin (5-HT) plays a role in the clinical effectiveness and mechanisms of action of SSRIs. However, the exact and complete mechanism of action and reasons for the low response rate to SSRIs in some adolescent populations remains unknown.

**Methods:**

To examine SSRI response and the role of 5-HT, this study will employ a randomised double-blind within subject, repeated measures design, recruiting adolescent patients with major depressive disorder. Participants will be subjected to acute tryptophan depletion (ATD) and the balanced control condition on two separate study days within a first study phase (Phase A), and the order in which these conditions (ATD/balanced control condition) occur will be random. This phase will be followed by Phase B, where participants will receive open label pharmacological treatment as usual with the SSRI fluoxetine and followed-up over a 12-week period.

**Discussion:**

ATD is a neurodietary method typically used to investigate the impact of lowered brain 5-HT synthesis on mood and behaviour. The major hypothesis of this study is that ATD will be negatively associated with mood and cognitive functioning, therefore reflecting individual serotonergic sensitivity and related depressive symptoms. Additionally, we expect the aforementioned effects of ATD administration on mood to predict clinical improvement with regard to overall depressive symptomatology 12 weeks into SSRI treatment.

**Trial registration:**

Australian and New Zealand Clinical Trials Registry (ANZCTR) ACTRN12616001561471. Registered on 11 November 2016.

**Electronic supplementary material:**

The online version of this article (10.1186/s13063-018-2791-4) contains supplementary material, which is available to authorized users.

## Background

The Second Australian Child and Adolescent Survey of Mental Health and Wellbeing reported that approximately 5% of adolescents aged between 12 and 17 met criteria for a major depressive disorder (MDD) [1]. One of the most common pharmacological treatments for MDD in adolescents is the use of serotonin reuptake inhibitors (SSRIs). SSRIs are known to increase the availability of the neurotransmitter serotonin (5-HT) in the brain, and this is thought to contribute to the antidepressant effects of the pharmacological agent. However, it must be noted that the complete mechanism of action of SSRIs is not fully understood. The use of SSRIs is also associated with a number of limitations, including a delay in treatment response of often several weeks, as well as non-response for up to 40% in clinical populations of adolescents [2]. These limitations, when combined, place adolescents at an increased risk of experiencing a prolonged period in which symptoms of depression are not fully addressed or remain undertreated, particularly if the individual does not respond to SSRI treatment.

Therefore, there is a need to better understand the predictors of pharmacological SSRI treatment response to avoid treatment delays, and to enable adequate and personalised treatment options for young people with MDD. Examination of the predictors of SSRI treatment response requires a mechanistic understanding of the role of brain 5-HT in mood regulation [3]. Serotonin challenge procedures provide a research design that can clarify the mechanisms of brain 5-HT. An example of such a challenge methodology is acute tryptophan depletion (ATD). This methodology has been used in adults to investigate central nervous serotonergic neurotransmission, and additionally, the role of central nervous 5-HT in affective disorders [4–7].

ATD is a neurodietary method used to lower brain 5-HT synthesis, and works on the premise that the essential amino acid tryptophan (TRP) is the physiological precursor of brain 5-HT synthesis. As brain 5-HT can only be synthesised through the availability of TRP in the central nervous system, the consumption of foods or beverages lacking in TRP leads to a respective decline in central nervous 5-HT synthesis. ATD involves the administration of an amino acid mixture lacking in TRP after an overnight protein fast. The administered amino acids compete with endogenous TRP on the uptake into the central nervous system over the blood–brain barrier, which leads to decreased substrate availability for central nervous 5-HT synthesis for a period of several (usually 5–7) hours. Additionally, the administered amino acids stimulate protein synthesis in the liver, which takes TRP from plasma stores and also contributes to depletion (for a detailed review, see Dingerkus et al. [8]).

ATD has been shown to decrease mood in depressed adults in remission, particularly those using antidepressants, but not in healthy controls [9]. Mood response to ATD reliably predicted depressive episodes during a follow-up year [10]. Additionally, ATD reversed antidepressant-induced remission in a large proportion of a study sample involving adults with depressive symptoms [11]. Given its history of temporarily reversing remission amongst treated depressed adults, it may be that ATD is particularly potent amongst those who are responsive to SSRIs.

The impact of ATD on mood has only been investigated in adults, mostly because safe and effective ATD methods targeting central nervous 5-HT synthesis in young people were only recently demonstrated [12–14]. In order to make ATD suitable for use in young people, it must account for body weight. A body weight-adapted ATD procedure, called Moja-De, has been validated in rodents [15] and humans [8], but has never been used to investigate mood-related antidepressant treatment response in adolescents with MDD.

### Study objectives and hypotheses

The first objective of this study is to investigate the effects of ATD as a physiologically induced short-term central nervous serotonergic deficit on mood in adolescents with MDD (Phase A of the study) prior to open label treatment initiation with SSRI fluoxetine (Phase B of the study). We expect that, in Phase A, ATD will have dysfunctional effects on mood, emotional face recognition, aspects of reversal learning, and attention-related cognitive parameters as assessed within a testing battery, when compared to a balanced control condition. Such results would reflect a 5-HT sensitivity to ATD with regards to the aforementioned parameters relevant for depression [16, 17], and would be in line with the findings amongst the adult population [10, 18–20].

The second objective of this study is to determine if the effects of ATD on mood as a physiological 5-HT-related neurochemical and dietary challenge procedure predicts treatment response to SSRI administration after a period of 12 weeks of open label pharmacological treatment with fluoxetine (Phase B). We hypothesise that the effects of ATD administration on mood from Phase A will predict clinical improvement relating to depressive symptoms and overall depressive symptomatology 12 weeks into SSRI treatment (Phase B). Specifically, we hypothesise that ATD may be indicative of serotonergic vulnerability in relation to symptoms relevant for adolescent depression.

### Trial design

In Phase A, this study will use a randomised, double-blind, within subject repeated measures design. During this phase, participants will undertake the challenge procedures (either ATD or balanced control condition (BAL)) on two separate study days, spaced at a maximum of 7 days apart. In Phase B, this study will employ an open label fluoxetine treatment for a period of 12 weeks.

## Methods

### Setting of the study

The present study will be conducted at Bentley Health Service, Perth, Australia.

### Subjects and eligibility criteria

This pilot study aims to recruit *N* = 20 participants, and all participants who are referred to the study will be screened. Inclusion criteria for this study are as follows: adolescents aged between 12 and 17 years with a confirmed diagnosis of MDD (ICD-10 or DSM-5; diagnosis to be confirmed through the administration of the Kiddie Schedule for Affective Disorders and Schizophrenia (K-SADS) [21], as well as a full psychiatric assessment), no current suicidal ideation or suicidal plans, no other psychiatric co-medications at study entry, IQ > 85 (screened through the use of the Wechsler Abbreviated Scale of Intelligence (WASI-II) [22]), and no active or past eating disorder. Exclusion criteria include current abuse of alcohol and use of illicit substances, pregnancy in female participants, chronic medical or neurological disorders, subjects whose primary language is not English, treatment with other histaminergic or dopaminergic medications, disorders of amino acid metabolism, and contraindications against the use of fluoxetine. Participation in this study is entirely voluntary. Once recruited, participants will be able to withdraw their consent at any time by informing the study team.

### Intervention

**Fig. 1:**
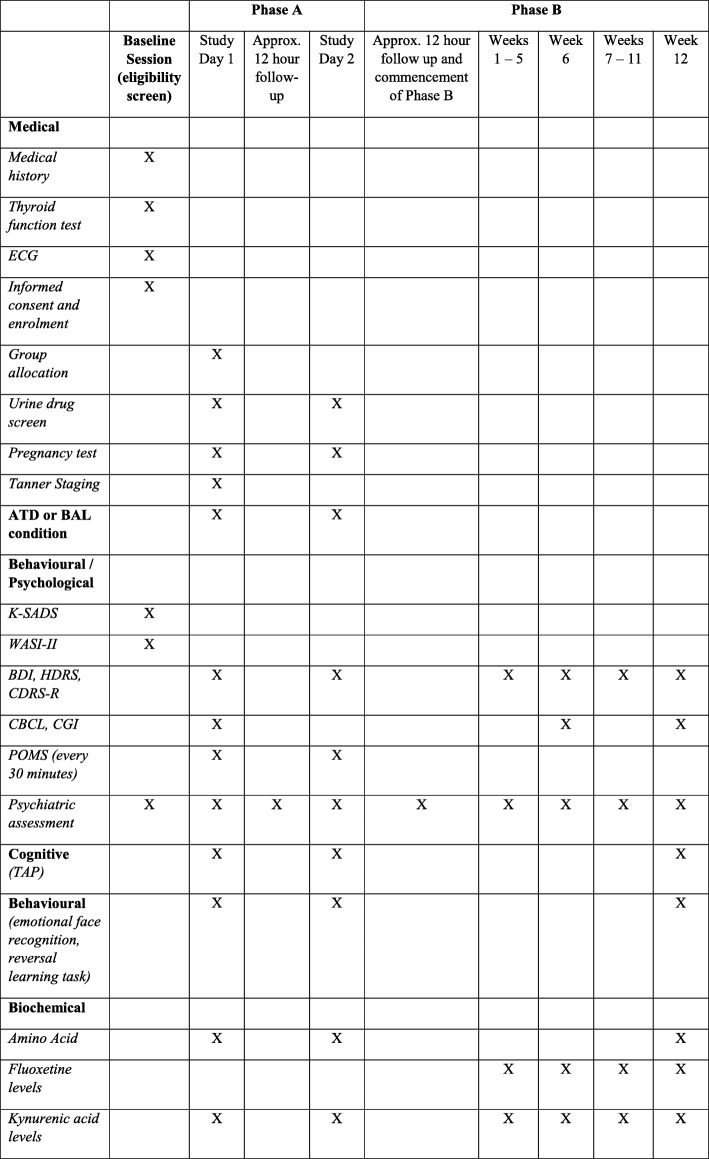
A schematic outline of the assessments that each participant will undertake during the TRACED study

This study will consist of one baseline session and two study phases, Phase A and Phase B. The timing of the assessments and study phases that will be conducted is illustrated in Fig. 1.

#### Phase A

Phase A will consist of two separate study days, where participants will be randomised to either receive the ATD (i.e. an amino acid beverage lacking TRP) or BAL (which will consist of an ATD-based amino beverage plus TRP). Each study day will be separated by a maximum of 7 days. For each study day of Phase A, participants will be required to undertake a drug and pregnancy screen (if female), will be administered the respective amino acid challenge beverages (ATD and BAL), and a series of psychological and cognitive assessments (see below). A total of seven blood samples will also be taken per study day in phase A. The type of assessments and rationale for the blood samples are further described below in more detail.

##### Amino acid challenge composition

The Moja-De ATD protocol (i.e. the ATD protocol that has been demonstrated to be safe for use in the child and adolescent population) employs amino acid administration within an aqueous suspension as a beverage, in which the relevant amino acid quantities are linked to the participant’s body weight. The amino acid quantities in ATD Moja-De are (dosage per 10 kg of body weight) as follows: L-phenylalanine (1.32 g), L-leucine (1.32 g), L-isoleucine (0.84 g), L-methionine (0.5 g), L-valine (0.96 g), L-threonine (0.6 g) and L-lysine (0.96 g). The respective control condition is known as the BAL condition. The BAL beverage contains the same amino acid quantities as described above for the ATD condition, but with an additional 0.7 g of TRP per 10 kg of body weight [23]. Research has shown that ATD is a safe and effective serotonergic challenge procedure [8, 15], and that it can be safely used in minors (Moja-De ATD test protocol) [12–14, 24].

Both the ATD and the BAL amino acid mixtures will be prepared by an approved manufacturing facility. The ATD amino acid beverage is made up of the seven dry amino acids as per the above calculations. Each subject receives a proportional amount of ATD amino acids within an aqueous suspension according to their body weight. For example, a 80 kg subject will receive 200 ml SyrSpend® SF (purified water modified food starch, sodium citrate, cinic acid, mali c acid, sodium benzoate, sulcralose, simethicone and cherry flavour) with the respective amino acids related to the individual’s body weight. As outlined above, the BAL beverage is made up of the same seven amino acids in the ATD with the addition of TRP (a total of eight amino acids) in the same way as the ATD beverage. Each subject receives a proportional amount of BAL amino acids within an aqueous suspension according to their body weight. Following the administration of both ATD/BAL beverages, apple juice will be offered following the mixture to wash out the taste.

##### Biochemical assessments and psychological and cognitive tasks to be conducted in Phase A

Several assessments and cognitive tasks will be conducted in Phase A, as outlined in Fig. 1. Assessments include the sampling of blood, mood assessments and cognitive testing.

The primary purpose of the blood samples collected at the beginning of Phase A is to assess baseline levels of TRP and other amino acids. The blood samples that will be collected during the two study days will allow measurement of the relevant amino acids contributing to the depletion. At the beginning of each study day in Phase A, participants will be required to complete the Beck Depression Inventory II (BDI-II) [25], the Hamilton Depression Rating Scale (HDRS) [26] and the Children’s Depression Rating Scale – Revised (CDRS-R) [27]. On the first study day, parents or caregivers will also complete the Child Behaviour Checklist [28]. Mood will be monitored on the two study days through the use of the Profile of Mood States (POMS) after ATD or BAL administration, in 30-min time intervals [29].

Cognitive assessments that will be conducted in Phase A include the Tests for Attentional Performance (TAP, PSYTEST, Herzogenrath, Germany) [30]. The TAP subtests that will be used include Alertness, Working Memory, Divided Attention and Sustained Attention. Between each test there will be a break of approximately 10 min. The Facial Expression Recognition Task will also be conducted on each study day of Phase A [31], as well as a Reversal Learning Task [32].

#### Phase B

Phase B will commence between 24 and up to a maximum of 72 h after the second day of Phase A. Phase B will consist of a 12-week open label pharmacological treatment as usual with the SSRI fluoxetine. Participants will be required to attend weekly follow-up sessions with the study doctors. Fluoxetine will be prescribed and administered in a fixed-dose approach, in accordance to the NICE Clinical Guidelines – Treatment of Depression in Children and Young People [33]. Specifically, the administration of fluoxetine will start at 10 mg daily and will be increased to 20 mg daily after 1 week.

During this second phase (Phase B), participants are free to participate in other treatments such as psychological treatment or psychotherapy (e.g. cognitive behavioural therapy) if medically indicated, and will not be excluded from the study unless the treatment with fluoxetine is stopped. However, these additional treatments will be taken into consideration with the statistical analysis and may lead to post-hoc exclusion of some participants. Under this approach, the data obtained will largely reflect the clinical reality in the sample served.

##### Biochemical assessments and psychological and cognitive tasks to be conducted in Phase B

Blood, mood and cognitive measurements will also be taken in Phase B. During Phase B, blood samples will be taken. The purpose of the blood testing during Phase B is to monitor fluoxetine availability throughout the course of the study. Fluoxetine levels assessed during this phase will also allow the calculation of preliminary dose–response relationships regarding the suggested predictors of treatment response. Compliance with the fluoxetine treatment will also be monitored through an empty packet return and verbal report. Blood samples will undergo basic laboratory processing before storage in an electronically lockable freezer at –80 °C degrees, before analysis. Participants’ mood will be measured on a weekly basis, using the BDI, HDRS, CDRS-R and POMS. Additionally, the Child Behaviour Checklist will be administered during the 6- and 12-week follow-up of Phase B to assess psychopathology. The cognitive tasks that were completed during each study day of Phase A will also be completed at the 12-week follow-up of Phase B. These tasks include the TAP, the Facial Expression Recognition Task and the Reversal Learning Task.

## Expected outcomes

### Mood

We expect that ATD-induced mood changes (Phase A) when compared to baseline and BAL administration will be inversely related to the mood-based treatment response to fluoxetine (as assessed via the POMS) and improvements in depressive symptomatology (BDI, HDRS, CDRS-R) 12 weeks into treatment (Phase B). In particular, our assumption is that symptom severity at baseline and change in mood following the ATD challenge will be proportional to mood change due to fluoxetine treatment as response to ATD may be an index of serotonergic vulnerability for mood changes.

### Cognitive parameters, emotional face recognition and reversal learning

We expect that ATD will be associated with an impairment of tests of divided and sustained attention, impaired working memory and alertness. Additionally, we expect an increased negative bias on the facial expression recognition task. Such findings would be in line with well-characterised components of the cognitive profile in depression. ATD will also be expected to enhance prediction of punishment in the used observational reversal learning task.

#### Recruitment

Participants will be recruited mainly via Headspace Services, which is a National Youth Mental Health Foundation that provides early intervention mental health services to 12–25 year olds, and Princess Margaret Hospital. Additionally, local paediatricians, developmental and health services, and general practitioners will participate in identifying suitable participants. Participants who discontinue from the study will be replaced and will be sourced by the same recruitment processes.

#### Blinding procedure and assignment of condition

The randomisation procedure will be managed by the Princess Margaret Hospital Clinical Trials Pharmacy (as of June 2018; Perth Children’s Hospital Clinical Trials Pharmacy). The amino acids will be coded via a number, and this data will then be fed into the randomisation software with the onsite researchers being blind to the challenge given (either ATD or BAL on the respective study day). In addition, the pharmacy will provide a sealed envelope with the coding for each mixture pair (i.e. the ATD/BAL mixture for each subject) and these envelopes will be kept at the study site in case this information is needed. Additional factors will be accounted for by the randomisation software, namely challenge order (ATD on day 1 & BAL on day 2 vs. BAL on day 1 & ATD on day 2), sex (males/females) and BMI (above vs. below the 50th age-adjusted sex-specific BMI percentile). For this study, unblinding is only permissible in the instance of an adverse event.

#### Data preparation and statistical analysis

The data that will be collected will consist of information relating to medical history, mood, scores on cognitive tasks and blood samples. All data, including blood samples will be de-identified. Codes for the depletion (ATD) and sham depletion (BAL) will be held in an envelope in a securely locked space in the unlikely event of an emergency that requires the code to be broken. Only researchers on the listed research team will have access to the final dataset.

To examine changes in response to ATD (mood, cognitive parameters and tasks, amino acid concentrations, etc.) as well as after treatment (mood symptoms, cognition, SSRI monitoring) a series of repeated measures ANOVAs will be conducted with time (baseline, ATD administration and 12 weeks into treatment) and challenge procedure (ATD/BAL administration) as within-subject factors. We will also evaluate for a significant effect of relevant baseline amino acid levels on the observed changes. If such a significant interaction is discovered, ANCOVAs will be performed with baseline values as covariates.

#### Power analysis

As this study is being conducted within a cohort of adolescents, we expect there to be possibly smaller effects [34], which could be due to reduced neurochemical effects, as well as difficulties with subjective mood ratings by the participants. For *N* = 20 participants, a power of 0.97 was calculated (ANOVA, effect for within-subjects factors) to detect small effects (effect sizes of 0.4) with a significance level of α = 0.05 (two-tailed testing, critical F = 3.26). This is in line with previous research showing that mood effects observed after ATD can be rather small in healthy subjects, but possibly more severe in patients affected or vulnerable to depression [35, 36]. Power calculations were performed with G*Power Software, Version 3.1.3 [37]. It is important to note, however, that these calculations are preliminary (conducted before study initiation) and are currently an estimate. The data of this study can be used to inform future larger scale studies. Individuals who are drop-outs or lost to follow-up will be replaced to meet the power calculations.

#### Trial management

This trial will be managed by the Centre & Discipline of Child and Adolescent Psychiatry, Psychosomatics and Psychotherapy and the Bentley Mental Health Service. The trial will employ several measures to ensure participant safety.

During Phase A, participants will consume the dietary protein amino acid serotonin depletion mixture (ATD challenge). This mixture may cause nausea and vomiting. Additionally, ATD is expected to decrease mood between 5 and 7 h (at maximum). To minimise associated risks with lowered mood, individuals with active suicidal ideation or plans will not be recruited to the study. A study doctor will be available at all times during Phase A to assess and manage these symptoms. Participants will be closely supervised and mood will be monitored frequently on the study days to identify those at risk. Mood will be formally assessed once per hour over 7 hours for each study day (Phase A). Tryptophan replacement (re-feeding) will be given at the end of each study day to normalise substrate availability for 5-HT synthesis. A thorough psychiatric assessment (i.e. mental state, risk assessment and management plan) will be conducted at the beginning and at the end of each study day to clear participants for discharge. The following day, participants will attend a clinical review by a study doctor to assess mood and risk. There will also be close monitoring in Phase B of the study, where participants will be reviewed by the study psychiatrists on a weekly basis for 12 weeks.

Any risk that emerges during the study (i.e. during Phase A or Phase B) will be managed by the research coordinator, who will liaise with the participant’s parent or guardian and arrange for prompt assessment by the participant’s study doctor or the nearest emergency department. All participants will have the research coordinator’s phone number, who will be available during working hours and after-hours of Phase A of the study, and during working hours of Phase B of the study. All participants will be given the Acute Response Team contact details for emergency after-hours support, which is an emergency community-based service that is available 24 h per day. The Acute Response Team will have been briefed about the rationale of this study and will also be regularly informed with regards to the number of participants involved at any point in time. Participants will be provided with a diary card with all relevant contact numbers.

A detailed log will be kept of any adverse events, and this log will be tracked by a Data and Safety Monitoring Committee. Members on this committee are independent of the research team.

The research group has also considered various common and serious adverse events that can occur in adolescents being treated for a major depressive episode with antidepressant medication. In everyday clinical practice, it can sometimes be difficult to be certain whether an adverse event is a result of a specific intervention or due to the depressive disorder. However, this trial will be paused and a review will be undertaken in the event of a completed suicide, suicide attempt, four individuals reporting an increase in acts of deliberate self-harm, three individuals reporting the development of hypomania, and two individuals reporting the development of psychotic–psychosis spectrum symptoms requiring hospitalisation or community-based treatment. Note that these numbers (based on incidence of events relative to percentage of the entire study sample) were estimated from one of the largest available cohorts of comparable patients, the Treatment of Adolescent Depression Study [38]. If patients meet exclusion criteria during the study but require further care, a care plan is in place where participants will have access to care in a community-based mental health setting.

#### Dissemination policy

The final report and publication will be submitted to a relevant academic journal, and findings will be presented in local and international conferences. Furthermore, a summary of the study and its findings will be made available for all participants.

## Discussion

This study may identify a new, simple dietary method to predict treatment response, leading to more targeted pharmacological treatment of adolescents with MDD, and may result in a more rapid treatment response. Children and adolescents may tolerate the ATD/BAL amino acid mixtures better than adults. There have been no serious side effects or complications reported in the studies conducted in minors [12–14, 39]. This study may identify a new, simple dietary method to predict SSRI-related treatment response in terms of serotonergic vulnerability, possibly leading to more targeted pharmacological treatment of adolescents with MDD. In turn, this may lead to a more rapid treatment response and better outcomes. Consequently, adolescents would be able to return to their prior level of functioning earlier, decreasing the overall burden of disease for the individual and allow better integration into the community. Such an approach needs to be confirmed by future larger-scale studies.

### Trial status

This manuscript is based on Protocol Version 11, dated 15th November 2017, and was prepared with reference to the SPIRIT Checklist (Additional file 1).

## Additional file

Additional file 1: SPIRIT 2013 Checklist: Recommended items to address in a clinical trial protocol and related documents*. (DOC 119 kb)

## Abbreviations

5-HT: serotonin
ATD: acute tryptophan depletion
BAL: balanced control condition
BDI: Beck Depression Inventory
CDRS-R: Children’s Depression Rating Scale - Revised
HDRS: Hamilton Depression Rating Scale
MDD: Major Depressive Disorder
POMS: Profile of Mood States
SSRI: Selective Serotonin Reuptake Inhibitors
TAP: Testbattery for Attentional Performance
TRP: L-tryptophan
